# Aging-associated mechanisms and metabolic vulnerabilities in esophageal carcinoma: an integrative review

**DOI:** 10.3389/fcell.2025.1714824

**Published:** 2025-12-16

**Authors:** Qiwei Liu, Zhiyu Xie, Wei Li, Mengxiang Li, Yijun Qi

**Affiliations:** 1 Key Laboratory of Cell Behavior, Medical School of Xuchang University, Xuchang, Henan, China; 2 College of Chemical and Materials Engineering, Xuchang University, Xuchang, Henan, China; 3 Department of Stomatology, Xuchang Central Hospital, Xuchang, Henan, China; 4 Department of Mathematics and Physics, Luoyang Institute of Science and Technology, Luoyang, Henan, China; 5 State Key Laboratory of Esophageal Cancer Prevention and Treatment, Henan Key Laboratory of Microbiome and Esophageal Cancer Prevention and Treatment, Henan Key Laboratory of Cancer Epigenetics, Cancer Hospital, The First Aûliated Hospital of Henan University of Science and Technology, Luoyang, Henan, China

**Keywords:** esophageal cancer, aging, metabolism, tumor microenvironment, senescence, immunosenescence

## Abstract

Esophageal carcinoma (EC), comprising esophageal squamous cell carcinoma (ESCC) and adenocarcinoma (EAC), remains a deadly malignancy predominantly affecting individuals over 60 years of age. Although advances in diagnosis and treatment have improved outcomes for some cancers, EC still carries a poor prognosis, particularly in the elderly. Aging contributes significantly to EC pathogenesis via accumulated genetic mutations, metabolic deregulation, immunosenescence, and a pro-tumorigenic microenvironment. In this review, we synthesize current understanding of the molecular alterations that drive EC, with particular emphasis on age-related factors. We examine key genomic mutations (e.g., TP53, chromosomal instability), metabolic reprogramming (including glycolysis, glutaminolysis, and lipid metabolism), and their interplay with the tumor microenvironment (TME). We also highlight the influence of aging processes such as clonal hematopoiesis, senescence-associated secretory phenotype (SASP), and immune exhaustion in modulating tumor behavior and therapeutic resistance. We propose that aging-associated metabolic and immunologic alterations represent promising targets for therapeutic intervention. Greater integration of aging biology into EC research and clinical strategy is needed to advance personalized care for elderly patients. This review provides a conceptual foundation for future translational and clinical studies at the intersection of oncology and aging.

## 1 Introduction

Esophageal cancer (EC) represents a significant global health challenge, ranking among the leading causes of cancer-related mortality worldwide. The two major histological subtypes, esophageal squamous cell carcinoma (ESCC) and esophageal adenocarcinoma (EAC), exhibit distinct epidemiological patterns. ESCC remains predominant in high-incidence regions such as East Asia and parts of Africa, largely influenced by environmental and lifestyle factors including tobacco use, alcohol consumption, and dietary habits (Chen et al., 2016). In contrast, EAC incidence has risen sharply in Western countries, correlating with the increasing prevalence of gastroesophageal reflux disease, obesity, and Barrett’s esophagus (Siegel et al., 2016). The disease is most prevalent among older adults, with a median age at diagnosis above 65 years (Liu C.‐Q. et al., 2022). Late-stage detection, aggressive biology, and resistance to therapy contribute to the dismal prognosis of EC.

Recent studies reveal that aging is not only a demographic risk factor but also a biological modifier of cancer progression (Trastus and Fagagna, 2025). Accumulated somatic mutations, epigenetic alterations, cellular senescence, and chronic low-grade inflammation are hallmarks of aging that intersect with cancer biology. In EC, aging shapes the tumor microenvironment (TME), metabolic landscape, and immune response, yet these connections remain underexplored.

In this review, we provide a mechanistic synthesis of genetic, metabolic, and aging-related factors in EC. We aim to highlight knowledge gaps and identify potential therapeutic targets for an aging EC population.

## 2 Genetic alterations of esophageal carcinoma

The development and progression of esophageal carcinoma are driven by a complex interplay of genetic, epigenetic, and environmental factors. While esophageal squamous cell carcinoma (ESCC) and esophageal adenocarcinoma (EAC) share some molecular features, each subtype possesses distinct alterations reflecting differences in cellular origin and environmental exposure (Table 1). Understanding these mechanisms is critical for identifying actionable targets and improving clinical management.

**TABLE 1:** Comparison of molecular characteristics between esophageal squamous cell carcinoma (ESCC) and esophageal adenocarcinoma (EAC).

Category	ESCC	EAC
Gene mutations	TP53 (90%–93%), CDKN2A/p16, NOTCH1-3, NFE2L2, PIK3CA, CCND1, SOX2, TP63, PTEN, RB1, FAT1-4, AJUBA, YAP1, FBXW7	TP53 (70%–80%), CDKN2A/p16, PIK3CA, KRAS, ERBB2, ARID1A, SMAD4
Epigenetic regulation	DNA methylation: CDKN2A, RASSF1A, FHIT histone modification: KMT2D, KMT2C, KDM6A, EP300, CREBBP Non-coding RNA regulation: miR-21, miR-200, miR-31, lncRNA-HOTAIR, lncRNA-PVT1	DNA methylation: CDKN2A, APC, MGMT Non-coding RNA regulation: miR-192, miR-194, miR-205, lncRNA-H19, lncRNA-MALAT1
Dysregulated signaling pathways	PI3K/AKT/mTOR, p53, NOTCH, NRF2-KEAP1, NF-κB/STAT3, Hippo	PI3K/AKT/mTOR, p53, Wnt/β-catenin, TGF-β/SMAD4, EGFR/ERBB2, RAS/MAPK
Chromosomal instability (CIN)	1p, 1q, 6p, 7q22, 8p, 10q, 11q, 12p11, 13q, 15q, 19q, 20q, 4p, 4q, 5q, 6q, 9p, 11p, 12q, 18q, 20p, 21q, 22q 2q, 5p, 8p,14q, 17q11, 17q25, 22q, 1p, 2q, 3p 8q24 (MYC), 9p21 (CDKN2A), 3q26 (SOX2), 11q13 (CCND1), 3p14 (FHIT)	1p, 1q, 6p, 7q22, 8p, 10q, 11q, 12p11, 13q, 15q, 19q, 20q, 4p, 4q, 5q, 6q, 9p, 11p, 12q, 18q, 20p, 21q, 22q 8q24 (MYC), 9p21 (CDKN2A), 17q12 (ERBB2), 18q21 (SMAD4)

Red color indicates the common molecular characteristics.

### 2.1 Mutational architecture of esophageal squamous cell carcinoma

Comprehensive genomic profiling studies have revealed that ESCC exhibits an exceptionally complex mutational architecture characterized by a high burden of somatic mutations, extensive copy number variations (CNVs), and structural chromosomal rearrangements that collectively drive malignant transformation and tumor progression. Among these alterations, TP53 stands out as the most frequently mutated gene, with loss-of-function mutations detected in approximately 90%–93% of cases, underscoring its role as a nearly universal driver event in squamous carcinogenesis (Lin et al., 2014; Song et al., 2014). These TP53 mutations often co-occur with loss of heterozygosity (LOH), further amplifying their oncogenic impact. Beyond TP53, the genomic landscape encompasses recurrent inactivation of multiple tumor suppressor genes, including CDKN2A (mutated in 8% and deleted in 47% of cases), PTEN, RB1, and NOTCH1-3, alongside frequent alterations in members of the FAT gene family (FAT1–FAT4), which regulate Hippo pathway signaling (Sawada et al., 2016; Liu et al., 2017).

Concurrently, oncogene activation serves as another major driver of ESCC pathogenesis. Amplifications and activating mutations of genes such as MYC, CCND1 (amplified in 46% of cases), and PIK3CA are recurrently observed, promoting aberrant cell cycle progression, enhanced proliferative signaling, and resistance to growth inhibition. Genome-wide analyses have identified recurrent copy number alterations in chromosomal regions 11q (harboring CCND1), 3q (SOX2), 2q (NFE2L2), and 9p (CDKN2A), which represent trunk mutations—early clonal events crucial for the initiation and clonal evolution of ESCC (Liu et al., 2017).

### 2.2 Epigenetic dysregulation and chromatin remodeling

Recent integrative genomic analyses have highlighted the pivotal role of epigenetic dysregulation in the pathogenesis of esophageal squamous cell carcinoma (ESCC). Aberrant DNA methylation frequently results in the silencing of key tumor suppressor genes such as CDKN2A, RASSF1A, and FHIT, thereby disrupting normal cell-cycle control and apoptosis (Guo et al., 2007; Mao et al., 2011). In parallel, mutations in epigenetic regulators—including KMT2D, EP300, KDM6A, and CREBBP—are recurrently observed, leading to perturbations in histone modification landscapes. These alterations collectively remodel the epigenomic architecture, producing abnormal gene expression programs that drive malignant transformation and tumor progression (Gao et al., 2014).

In addition to DNA and histone modifications, non-coding RNA (ncRNA)-mediated regulation plays a crucial role in ESCC progression. Dysregulated expression of miR-21, miR-200, and miR-31, as well as long non-coding RNAs (lncRNA-HOTAIR and lncRNA-PVT1), has been shown to promote tumor cell invasion, migration, and epithelial–mesenchymal transition, underscoring the multifaceted regulatory network orchestrated by ncRNAs (Feng et al., 2020).

The convergence of genetic and epigenetic alterations is exemplified by pathway-level dysregulation affecting critical developmental and tumor suppressor networks. Disruptions in Hippo signaling through alterations in FAT1-4, AJUBA, and YAP1, combined with Notch pathway inactivation via mutations in NOTCH1-3 and FBXW7, collectively orchestrate the loss of growth control and differentiation that characterizes ESCC (Agrawal et al., 2012; The Cancer Genome Atlas Research Network, 2017).

### 2.3 Pre-malignant field effects and clonal evolution

A particularly intriguing aspect of ESCC biology is the observation that histologically normal esophageal epithelium frequently harbors substantial somatic mutation burdens, many of which overlap with alterations found in invasive carcinomas (Martincorena et al., 2018; Yokoyama et al., 2019). This phenomenon of “field cancerization” complicates the delineation between initiating driver events and passenger mutations acquired during clonal evolution. Longitudinal studies have demonstrated that TP53 mutations followed by LOH events serve as key drivers of clonal expansion and genomic instability, facilitating the transition from pre-malignant to malignant states. Paradoxically, while NOTCH1 mutations are prevalent in the normal human esophageal epithelium by middle age, emerging evidence suggests they may exert context-dependent tumor suppressive effects by constraining malignant progression despite promoting clonal competition within the epithelial compartment (Abby et al., 2023).

### 2.4 Mutational architecture of esophageal adenocarcinoma

In contrast to ESCC, EC exhibits a fundamentally distinct mutational spectrum that reflects its different cellular origins and pathogenetic mechanisms. EAC is characterized by TP53 mutations in 70%–80% of cases, followed by recurrent alterations in CDKN2A, PIK3CA, KRAS, ERBB2, ARID1A, and SMAD4. Notably, EAC demonstrates a high propensity for genomic catastrophes, with chromothriptic events occurring in approximately 32% of cases, contributing to the remarkable genomic complexity that characterizes this malignancy (Hoppe et al., 2021; Yasar, 2023).

The molecular alterations in EAC demonstrate significant overlap with gastric and colorectal adenocarcinomas, reflecting shared oncogenic pathways among gastrointestinal tract adenocarcinomas. ERBB2 amplification, detected in approximately 15%–20% of EAC cases, enhances proliferative and survival signaling through PI3K/AKT and MAPK pathway activation (Dahlberg et al., 2004; Augustin et al., 2022). SMAD4 loss, which occurs in a substantial subset of EACs, promotes chromosomal instability and disrupts TGF-β-mediated growth inhibitory signaling and has been associated with increased propensity for disease recurrence and poor survival outcomes (Gotovac et al., 2021). Similarly, KRAS activating mutations promote tumorigenesis through constitutive activation of RAS/MAPK signaling cascades, the consequences include enhanced cellular proliferation, resistance to growth inhibitory signals, evasion of apoptosis, and promotion of angiogenesis and invasive capabilities (Huang et al., 2021).

The accumulating evidence of genetic and pathway-level divergences between ESCC and EAC underscores the critical importance of subtype-specific molecular profiling in clinical practice. Advanced computational approaches integrating multi-omics data are revealing novel therapeutic vulnerabilities and predictive biomarkers. Continued functional characterization of identified alterations, particularly those affecting epigenetic regulatory networks and metabolic pathways, will be essential for translating genomic discoveries into clinically actionable therapeutic strategies. The development of patient-derived models and organoid systems will facilitate the validation of these findings and accelerate the development of personalized treatment approaches for these aggressive malignancies.

### 2.5 Chromosomal instability of esophageal carcinoma

Chromosomal instability (CIN) represents a defining feature of both ESCC and EAC, underpinning their extensive tumor heterogeneity and therapeutic resistance. Approximately two-thirds of esophageal carcinomas exhibit high genomic instability (Hu et al., 2009).

Widespread CNVs are observed across both subtypes but display distinct patterns. Frequent gains of chromosome 1p, 1q, 6p, 7q22, 8p, 10q, 11q, 12p11, 13q, 15q, 19q, and 20q and loss of chromosome 4p, 4q, 5q, 6q, 9p, 11p, 12q, 18q, 20p, 21q, and 22q occur in both ESCC and EAC, but gains of 2q, 5p, 8p,14q, 17q11, 17q25 and 22q are mainly seen in ESCC, and loss of chromosome 1p, 2q and 3p is primarily observed in ESCC. Notably, chromosome 14 amplification occurs in 35% of ESCC cases but only 4% of EAC (Bandla et al., 2012).

Oncogene amplification constitutes a major mechanism of oncogenic activation. Amplifications of CCND1, SOX2, and TP63 are recurrent in ESCC, whereas ERBB2, VEGFA, GATA4, and GATA6 are preferentially amplified in EAC (The Cancer Genome Atlas Research Network, 2017; Jiang et al., 2023). MYC amplification is common in both subtypes and contributes to proliferation and self-renewal of esophageal epithelial cells (Lian et al., 2017; Huang et al., 2019; Shi et al., 2020; Hishida et al., 2022). Recent evidence indicates that extrachromosomal DNA and breakage–fusion–bridge cycles promote recurrent amplifications of MYC, ERBB2, MDM2, and HMGA2 in EAC (Ng et al., 2024).

Conversely, deletions of tumor suppressor genes such as CDKN2A and FHIT are frequent events. Loss of CDKN2A at the 9p21 locus, often co-occurring with promoter hypermethylation, leads to cell-cycle deregulation and poor prognosis in EAC (Ito et al., 2007; Zhou et al., 2017; Ganguli et al., 2025). Similarly, FHIT deletion disrupts genomic integrity and enhances tumor progression. The coexistence of deletions and epigenetic silencing in key regulatory genes exemplifies a “dual-hit” mechanism that reinforces tumor suppressor inactivation. Together, these alterations reflect a synergistic interplay between genetic and epigenetic dysregulation, shaping the complex molecular landscape that drives esophageal carcinogenesis.

## 3 Tumor microenvironment of esophageal carcinoma

The tumor microenvironment (TME) plays a central role in the initiation, progression, and therapeutic response of EC. It comprises a dynamic ecosystem of cancer-associated fibroblasts (CAFs), immune and endothelial cells, extracellular matrix (ECM), and soluble mediators such as cytokines and growth factors. Within this complex milieu, reciprocal interactions between malignant and stromal cells dictate tumor behavior, immune evasion, and therapy resistance (Lin et al., 2016; Khan et al., 2024). Various molecules released by stromal components facilitate tumor growth through direct activation of cancer cell growth signals and extensive microenvironmental remodeling. Recent advances in single cell sequencing technologies have greatly deepened our understanding of the complex cellular landscape of esophageal cancer.

The immune landscape of the EC TME is predominantly immunosuppressive. Tumor-associated macrophages (TAMs), particularly those of the M2-like phenotype, play a central role in dampening cytotoxic T cell responses and facilitating metastasis. Emerging evidence suggests that TAMs may transition from pro-inflammatory M1 to immunosuppressive M2 states, potentially driven by Notch1 mutation signaling (Yang et al., 2023). In addition to TAMs, regulatory T cells (Tregs) and myeloid-derived suppressor cells (MDSCs) contribute to immune evasion through distinct but complementary mechanisms. Elevated infiltration of MDSCs has been associated with enhanced tumor growth in preclinical models and correlates with poor prognosis in EC patients, underscoring their oncogenic potential within the TME (Gabitass et al., 2011). Furthermore, upregulation of immune checkpoint molecules, such as PD-L1, is frequently observed in both EAC and ESCC and is linked to unfavorable clinical outcomes (Yu et al., 2016). This immunosuppressive milieu, however, provides a rationale for therapeutic strategies targeting immune checkpoint pathways such as anti-PD-1/PD-L1 Immunotherapy (Chen K. et al., 2021; Janjigian et al., 2021; Yoon et al., 2022).

Upon initial activation, immune effector cells including dendritic cells (DCs), effector T cells (T effs), memory T cells (T mems), and natural killer cells (NKs) mount coordinated immune responses against tumor cells within the TME, thereby controlling tumor progression and preventing immune surveillance evasion (Ji et al., 2024). During the tumor progression, the immune cell populations changed overtime, for example, in the early stages of ESCC, non-tumor regions are predominantly enriched with naïve CD4^+^ T cells, memory CD4^+^ T cells, and Th17 cells, whereas tumor regions are characterized by an increased presence of regulatory T cells (Tregs), cytotoxic T cells, and pre-terminal exhausted T cells (Yao et al., 2020; Zheng et al., 2020; Chen et al., 2021b; Dinh et al., 2021). As the disease advances, there is a notable decline in the proportions of naïve and memory CD4^+^ T cells within non-tumor areas, accompanied by a progressive accumulation of exhausted CD4^+^ and CD8^+^ T cells. Concurrently, the proportion of cytotoxic T cells within tumor regions diminishes.

### 3.1 Hypoxia and VEGF signaling

Hypoxia is a prominent feature of the EC TME due to abnormal vascularization. Hypoxic stress induces stabilization of hypoxia-inducible factor 1α (HIF-1α), which transcriptionally activates genes involved in angiogenesis (e.g., VEGF), glycolysis, and cell survival (Chen Z. et al., 2023). Hypoxia promotes cancer progression by inducing lactylation of Serine hydroxymethyl transferase 2 (SHMT2) or Axin1 protein to promote glycolysis of EC cells (Li et al., 2024; Qiao et al., 2024), or by introducing a novel hypoxia induced long noncoding RNA (lncRNA), lnc191 as a key driver activating GRP78/ERK/MAPK signaling in the ESCC progression (Wei et al., 2024). Hypoxia supports cell survival via reducing hypoxia-induced apoptosis by inhibiting the GRP78-perk-eIF2α-ATF4-CHOP pathway (Lin et al., 2023), or by inhibiting ferroptosis in ESCC via the USP2-NCOA4 pathway (Song et al., 2024). A recent study identifies a hypoxia induced senescent cancer associated fibroblast (hsCAF) subpopulation that promotes ESCC stemness and chemoresistance via IGF1 signaling, highlighting hsCAFs as a potential therapeutic target (Ou et al., 2025). Hypoxia also promotes resistance to radiotherapy and chemotherapy (Zhu Z. J. et al., 2021). Currently identifying hypoxia responsive molecular markers is key to figure out personal response to therapy in EC (Peerlings et al., 2017; Xiao et al., 2022), such as VBP1, a key gene in the hypoxia-related prognostic signature, drives ESCC tumor proliferation (Miao et al., 2024).

In EC, hypoxia tumor environment stimulates vascular endothelial growth factor (VEGF) expression, which plays a pivotal role in tumor progression by promoting angiogenesis, a process of formation of new blood vessels, supporting tumor growth by supplying essential nutrients (Yang et al., 2020). Previous studies have established positive correlations between serum VEGF levels and tumor staging, with elevated VEGF content correlating with poor prognosis ESCC patients (Shimada et al., 2001).

Furthermore, angiogenesis driven by VEGF and other pro-angiogenic signals supports tumor growth and dissemination. However, the resulting vasculature is often irregular and leaky, exacerbating hypoxia and drug delivery barriers, which requires further investigation. Besides chemotherapy, treatments have also focused on targeting HER-2, PD-1, and novel targets like VEGF to improve outcomes (Siddiqui and Almhanna, 2021).

### 3.2 Cancer-associated fibroblasts (CAFs): recent advances

CAFs represent a heterogeneous population that promotes cancer cell proliferation, migration, invasion, immunosuppression, and therapeutic resistance in solid tumors through secretion of cytokines and growth factors, extracellular matrix (ECM) remodeling, and metabolic support provision (Wilczyński et al., 2024), CAFs are characterized by upregulation of all collogen genes when compared to normal fibroblasts (Chen et al., 2021b). Recent research has significantly advanced our understanding of CAF biology in EC (Xue et al., 2023).

Current findings indicate that during disease progression, inhibition of transcription factor KLF4 in epithelial cells results in marked reduction of ANXA1 expression, which serves as a ligand for formyl peptide receptor type 2 (FPR2). This ANXA1 decrease triggers unregulated conversion of normal fibroblasts into CAFs, facilitating tumor-stroma crosstalk (Chen Y. et al., 2023). This advance has confirmed that precancerous esophageal epithelial cells can reprogram normal resident fibroblasts into CAFs by downregulating the ANXA1-FRP2 signaling pathway (Liu et al., 2019a).

CAFs have emerged as major focuses for targeted therapy development in EC, with enhanced understanding of their role in treatment resistance (Lan et al., 2025). Recent studies have identified periostin as a key factor in CAF-mediated ESCC progression, where periostin in CAFs enhances both cancer and stromal cell migration (Miyako et al., 2024). Mechanistic studies demonstrate that periostin derived from CAFs promotes ESCC progression through ADAM17 activation, providing new therapeutic targets (Ishibashi et al., 2023). Additionally, cancer-derived S100A8 engages with CD147 receptors on CAFs, triggering their polarization and fostering chemoresistance via activation of the intracellular RhoA-ROCK-MLC2-MRTF-A signaling pathway (Liu et al., 2019b).

Recent advances include high-plex protein and whole transcriptome co-mapping at cellular resolution with spatial CITE-seq technology, enabling detailed characterization of TME components. These technological advances are providing unprecedented insights into TME complexity and offering new therapeutic targets. The evolving understanding of esophageal cancer TME emphasizes the need for personalized approaches that consider the specific cellular and molecular composition of individual tumors, paving the way for more effective precision medicine strategies.

## 4 Metabolic alterations in esophageal carcinoma

EC cells like broader cancer types undergo profound metabolic reprogramming to support their uncontrolled growth and survival in nutrient-limited tumor microenvironments (Heiden and DeBerardinis, 2017; Faubert et al., 2020; Dong et al., 2025). Metabolic heterogeneity in EC is a critical factor contributing to poor clinical outcomes, arising from the complex interplay between the tumor microenvironment and genetic factors of tumor cells (Guo et al., 2025). Metabolic reprogramming of cancer cells, including increased glycolysis (the Warburg effect), elevated glutaminolysis, and dysregulated lipid metabolism, contributes to an immunosuppressive tumor microenvironment and resistance to cancer immunotherapy (Shende et al., 2025).

### 4.1 The Warburg effect and glycolytic reprogramming

One of the hallmark alterations is aerobic glycolysis, also known as the Warburg effect, characterized by enhanced glucose uptake and lactate production even under normoxic conditions (Warburg, 1956). The Warburg effect, characterized by the preferential conversion of glucose to lactate even in the presence of oxygen and functional mitochondria, is a prominent metabolic hallmark of cancer cells and has emerged as a promising therapeutic target for cancer therapy (Barba et al., 2024).

This metabolic shift is driven by oncogenic activation of pathways such as PI3K/Akt/mTOR and MYC, leading to the upregulation of glycolytic enzymes including hexokinase 2 (HK2) and lactate dehydrogenase A (LDHA) (Martinez-Outschoorn et al., 2016). Recent studies emphasize that energy–metabolic differences are easily found in the growth, invasion, immune escape and anti-tumor drug resistance of cancer cells (Fukushi et al., 2022). Clinically, increased Fluorodeoxyglucose (FDG) uptake on PET-CT scans reflects this metabolic phenotype and is used in EC diagnosis and prognosis (Cremonesi et al., 2017).

Beyond its role in energy production, aerobic glycolysis profoundly shapes the tumor microenvironment (TME) in EC (Reina-Campos et al., 2017; Zhu L. et al., 2021; Zhao et al., 2024). High glycolytic activity in tumor cells not only increases glucose consumption and lactate production, but also creates a hypoxic and acidic TME (Pérez-Tomás and Pérez-Guillén, 2020). Hypoxia stabilizes hypoxia-inducible factor 1α (HIF-1α), a key regulator that enhances glycolytic gene expression and promotes tumor survival via the PI3K/Akt/mTOR and NF-κB pathways (Majumder et al., 2004; Luo et al., 2022). Enzymes involved in pyruvate metabolism such as PKM2 (Ma et al., 2019), PDK (Palsson-McDermott et al., 2015) and LDHA (Chen et al., 2025) are upregulated by HIF-1α in EC, supporting metabolic reprogramming towards more lactate production and acidic TME, as a result correlating with poor prognosis in EC (Semenza et al., 1994). This lactate-rich, glucose-deprived environment impairs anti-tumor immunity. It promotes M2-like polarization of TAMs, enhancing immunosuppressive functions and the secretion of pro-tumorigenic factors like VEGF, ARG-1, and CXCL12 (Colegio et al., 2014; Vitale et al., 2019). Of note, lactate further induces M2-like polarization of TAMs by stabilizing HIF-1α and activating STAT3 and ERK pathways (Mu et al., 2018). TAMs also contribute to nutrient support and angiogenesis within the TME (Chen et al., 2011).

T cells are especially sensitive to glucose availability. Glycolysis in tumor cells and TAMs depletes glucose, suppressing T cell function and promoting regulatory Treg differentiation (Chang et al., 2015; Kurniawan et al., 2020). Additionally, elevated TGF-β expression in CAFs, particularly after chemotherapy, promotes immune tolerance by activating Tregs and suppressing the function of effector T cells, NK cells, and dendritic cells (Zhang et al., 2016; Blum et al., 2019; Kim et al., 2021). Furthermore, CAFs secreted IL-6, another cytokine enriched in EC TME, drives epithelial-to-mesenchymal (EMT) transition, tumor stemness, and chemoresistance (Suchi et al., 2011; Zhao et al., 2015; Vinocha et al., 2018; Ebbing et al., 2019).

Taken together, glycolytic reprogramming in EC fosters a hostile TME that promotes tumor progression and suppresses immune responses, offering critical metabolic targets for immunotherapy for EC (Bader et al., 2020; Liu X. et al., 2022). Meanwhile, targeted therapies against key glycolytic enzymes have been developed (Table 2). Lonidamine inhibits the activity of HK2, blocking the conversion of glucose to lactate, thereby impairing tumor energy supply and biosynthesis. It has entered phase I clinical trials for solid tumors, including esophageal cancer (Nath et al., 2016; Guo et al., 2023). 2-Deoxy-D-glucose (2-DG) directly inhibits the glycolytic pathway, disrupting cancer cell energy production and biosynthesis. It has been evaluated in phase I clinical studies for solid tumors, though direct research in esophageal cancer remains limited (Pajak et al., 2020).

**TABLE 2:** Current clinical trial progress targeting metabolic reprogramming.

Drug	Mechanism	Phase	Relevance to EC
2-Deoxy-D-Glucose (2-DG)	Inhibit glycolysis (block HK2)	Phase I dose escalation, multi-cancer solid tumor combination trial	Specifically targeting glycolysis, it is suitable for the metabolic modification research of EC, but there is still a lack of studies specifically targeting EC
Lonidamine	Inhibits the activity of the key glycolytic enzyme HK2	Multiple phase I–II clinical trials	Phase I study targeting solid tumors (including EC)
CB839	Inhibit glutaminase (GLS)	Multiple phase I/II “basket” and combination studies; combinations with immunotherapy/targeted drugs are being evaluated	Although mainly studied in other solid tumors, glutamine metabolism has also been reported in EC, and it has the potential to migrate to EC
DRP-104	Broad-spectrum interference with glutamate/glutamine metabolism and activation of antitumor immunity	Phase I/Early clinical; early cohort and dose exploration for advanced solid tumors	Broad-spectrum inhibition of glutamate metabolism can simultaneously inhibit the metabolism of tumor cells and immune cells; it has potential value for metabolic-dependent EC subtypes (such as high glutamine metabolism)
V-9302	Competitively inhibits the glutamine transporter ASCT2 to block the uptake of glutamine by tumor cells	Preclinical research stage	It has potential value for metabolic-dependent EC subtypes (such as high glutamine metabolism)
TVB-2640	Inhibit fatty acid synthase (FASN)	Phase I has been completed and multiple phase II combination trials have been initiated	Abnormal lipid metabolism was also mentioned in EC. TVB-2640 is currently one of the most mature lipid metabolism-targeting drugs

### 4.2 Glutamine metabolism and anabolic support

In addition to glycolysis, glutamine metabolism is extensively rewired in EC to support anabolic processes and redox homeostasis (Cluntun et al., 2017). Glutaminase (GLS) overexpression facilitates glutamine catabolism to fuel the TCA cycle and generate NADPH, which helps counteract oxidative stress (Altman et al., 2016). Targeting GLS has shown promising preclinical efficacy in variety of cancer models (Meric-Bernstam et al., 2019; Jin et al., 2020; Wang Z. et al., 2020; Shen et al., 2021). GLS inhibitors, such as CB839, represent a promising therapeutic strategy in EC by targeting glutamine metabolism (Qie et al., 2019). CB839 not only suppresses tumor proliferation but also enhances the efficacy of immunotherapies like CAR-T cells (Varghese et al., 2021). In addition, glutamine transporter inhibitors, such as V-9302, further disrupt glutamine uptake in cancer cells, thereby augmenting the potential of glutamine-targeted therapies (Schulte et al., 2018; Yang et al., 2021). The glutamine antagonist DRP-104 also inhibits tumor growth through broad interference with glutamine metabolism (Yokoyama et al., 2022; Pillai et al., 2024) (Table 2).

Glutamine metabolic reprogramming is regulated by key oncogenes and tumor suppressors. MYC enhances glutamine utilization by directly activating glutamine metabolism genes or by suppressing miR-23a/b expression, which inhibits GLS (Wise et al., 2008; Gao et al., 2009). In contrast, p53 promotes oxidative metabolism and upregulates the GLS isoform GLS2, thereby limiting tumor growth (Hu et al., 2010). Other factors such as glutamate dehydrogenase 2 (GDH2), Isocitrate dehydrogenase 1/2 (IDH1/2), PI3K, STAT1, ERK, and KRAS also contribute to glutamine metabolic remodeling (Borodovsky et al., 2012; Thangavelu et al., 2012; Son et al., 2013; Liu et al., 2014; Takeuchi et al., 2018; Kondo et al., 2020).

Glutamine availability serves as a crucial regulator of essential cellular signaling networks. Research in porcine esophageal cells demonstrates that glutamine depletion suppresses both mTOR and MAPK/ERK pathways, whereas glutamine supplementation restores their activity (Zhu et al., 2014b). This amino acid functions not only as a growth promoter for malignant cells but also as a fundamental requirement for immune system function. Glutamine metabolism differentially regulates T cell fate and function, with GLS being essential for Th17 cell differentiation while limiting Th1 and CTL effector cell development (Johnson et al., 2018). CD8^+^ T cells require glutamine as an indispensable metabolic fuel, and when deprived of this nutrient, these cells experience permanent deficits in both proliferation capacity and cytokine secretion (Ma et al., 2024). Within the TME, malignant cells and T cells engage in metabolic competition for glutamine, frequently resulting in T cell glutamine starvation and compromised immune surveillance (Edwards et al., 2021). Consequently, therapeutic targeting of glutamine metabolism specifically in EC presents a dual-benefit strategy that can simultaneously restrict tumor progression and enhance anti-tumor immune responses (Wang B. et al., 2024).

Given the critical role of glutamine metabolism in both tumor growth and immune regulation, future therapeutic strategies in EC should focus on selectively targeting glutamine pathways to disrupt tumor metabolic demands while preserving or enhancing immune cell function. Glutaminase inhibitors such as CB839, glutamine transporter blockers such as V-9302, and broad-spectrum glutamine metabolism inhibitors such as DRP-104 have shown promising preclinical efficacy, highlighting the potential of combining metabolic therapy with immunotherapy (e.g., CAR-T cells) to overcome resistance and improve patient outcomes (Table 2). Further research is needed to refine targeting specificity, minimize off-target effects, and identify biomarkers that predict responsiveness to glutamine-targeted interventions in EC.

### 4.3 Lipid metabolism dysregulation

Recent studies have implicated significant lipid metabolism dysregulation in EC progression (Snaebjornsson et al., 2020; Jiao et al., 2024). Elevated fatty acid synthase (FASN) and stearoyl-CoA desaturase 1 (SCD1) levels have been observed in EC tissues and correlate with poor prognosis (Zhou et al., 2012; Vanauberg et al., 2023; Wang B. Y. et al., 2023). Lipid droplet accumulation may contribute to cellular stress resistance and metastatic potential, providing cancer cells with energy reserves and membrane building blocks for rapid proliferation (Cruz et al., 2020).

Altered lipid metabolism is a hallmark of ESCC, contributing to tumor progression, survival, and immune evasion within the TME (Jiao et al., 2024). Both lipid synthesis, uptake and fatty acid oxidation (FAO) are reprogrammed in ESCC, offering multiple therapeutic targets.

ESCC cells display enhanced lipid synthesis to meet the metabolic demands of rapid proliferation (Cheng et al., 2018). Key enzymes involved in this process include ATP citrate lyase (ACLY), FASN, and Lysophosphatidylcholine acyltransferase 1 (LPCAT1), regulated by the transcription factor sterol regulatory element-binding protein 1 (SREBP1). ACLY catalyzes a rate-limiting step in *de novo* lipid synthesis, and its overexpression promotes tumor cell proliferation, whereas its inhibition impairs growth (Granchi, 2018). FASN catalyzes the final step of fatty acid production and is minimally expressed in normal cells but upregulated in ESCC, where it supports tumor survival and is associated with poor prognosis (Zhou et al., 2012; Fhu and Ali, 2020). LPCAT1 regulates cholesterol metabolism, promoting tumor progression through PI3K/EGFR signaling mediated activation of SREBP1, and suggesting that LPCAT1 is a key factor in cancer lipid synthesis (Tao et al., 2021). Additionally, pre-mRNA processing factor 19 (PRP19) promotes ESCC progression by stabilizing SREBP1 mRNA in an m6A-dependent manner, thereby reprogramming fatty acid metabolism, this indicates PRP19 as a potential prognostic biomarker and therapeutic target (Zhang et al., 2023). All together, these studies suggest that SREBP1 is a central factor transcriptionally regulating ESCC cancer progression, SREBP1 overexpression promotes ESCC progression by enhancing fatty acid biosynthesis, while its silencing suppresses tumor cell proliferation, migration, and invasion, highlighting SREBP1 as key therapeutic target (Wang J. et al., 2020). To date, clinical trials targeting lipid metabolism have focused on FASN inhibitors, particularly TVB-2640, one of the most advanced lipid metabolism-targeted drugs, which effectively suppresses tumor growth (Table 2).

In hypoxic and acidic TME, lipid uptake is also elevated. Hypoxia-inducible factor 1α (HIF-1α) upregulates lipid transporters such as CD36, FABP3/7, low-density lipoprotein receptor (LDLR) and FA transport proteins (FATPs), promoting fatty acid influx and accumulation, which enhances the survival of tumor cells (Guo et al., 2011; Bensaad et al., 2014; Pascual et al., 2016; Veglia et al., 2019; Aoki et al., 2022). Preclinical studies have shown that blocking CD36, especially in combination with FASN inhibitors and anti-PD-1 therapy, produces synergistic anti-tumor effects, suggesting a promising combinatorial treatment strategy (Drury et al., 2020).

FAO, a parallel pathway to lipid synthesis, is also upregulated in ESCC. Carnitine palmitoyltransferase 1A (CPT1A), the rate-limiting enzyme of FAO, mediates fatty acid transport into mitochondria for β-oxidation via acylcarnitines, is frequently overexpressed in ESCC and correlates with advanced clinical stage, metastasis, and poor prognosis (Tian et al., 2022; Nandi et al., 2024; Zhang et al., 2025). STING suppresses ESCC progression by promoting CPT1A degradation and inhibiting FAO, and STING is downregulated in ESCC, suggesting that STING is a therapeutic target and prognostic marker for ESCC (Zhang et al., 2025). FAO supports redox homeostasis by generating NADPH and glutathione, thereby preventing apoptosis (Tian et al., 2022). Inhibition of CPT1A impairs anchorage-independent tumor growth and reduces NADPH supply, disrupting tumor cell survival. Furthermore, altered β-oxidation is reflected in the reduced plasma levels of medium- and long-chain acylcarnitines in ESCC patients (Wang et al., 2022), suggesting a dysregulated FAO could be a target for therapy.

Lipid metabolism also significantly influences immune cell function. The same metabolic pathways that support tumor growth can suppress anti-tumor immunity. For instance, CD36-mediated lipid uptake and CPT1A-driven FAO in TAMs promote their polarization toward the immunosuppressive M2 phenotype, enhancing angiogenesis and dampening immune responses (Su et al., 2020). Inhibition of mitochondrial FAO in TAMs reduces their pro-tumor activity and supports immune reactivation. Similarly, T cells rely on FAO for energy, particularly under nutrient-deprived conditions in the TME. However, the metabolic demands of tumor cells restrict nutrient availability, leading to impaired T cell activation and function (Tang et al., 2024). CD36-mediated lipid uptake in CD8^+^ T cells lead to lipid peroxidation, ferroptosis, and T cell exhaustion, impairing antitumor immunity. Targeting CD36 or ferroptosis can restore T cell function and improve immune checkpoint inhibitor efficacy (Liu et al., 2021; Ma et al., 2021; Wang R. et al., 2023).

The increased intake, synthesis, and oxidative utilization of lipids by tumor cells not only meet their energy demands but also reshape the metabolic landscape of the TME (Yu et al., 2021). This metabolic competition between tumor and immune cells leads to immunosuppression, immune escape, and therapy resistance (Cui et al., 2022). Integrating current research on both anabolic and catabolic aspects of lipid metabolism has highlighted key molecular targets such as FASN, SREBP1, CD36, and CPT1A. Targeting these pathways may disrupt tumor metabolism while simultaneously enhancing anti-tumor immunity, offering new directions for therapeutic intervention in ESCC.

## 5 Aging-related factors in esophageal cancer

Cancer is an age-related disease and is increasingly becoming the primary cause of mortality in the elderly population (Trastus and Fagagna, 2025). Aging is also a significant risk factor for EC, with most patients diagnosed over the age of 50 and an average age of ∼65 years. Despite declining incidence and mortality rates in China from 1990 to 2020, the absolute number of new EC cases and deaths continues to rise due to population aging. Projections estimate over 324,000 new cases and 416,000 deaths by 2030, increasing further by 2040, highlighting the growing burden of EC in an aging population (Liu C.-Q. et al., 2022; Zhu et al., 2023). The relationship between aging and EC represents a complex interplay of accumulated somatic mutation, cellular senescence, metabolic dysfunction, and immune system deterioration that significantly impacts cancer development, progression, and treatment outcomes.

### 5.1 Aging-related accumulated somatic mutation

Somatic mutations accumulate with age, primarily due to intrinsic mutational processes. Analysis of normal esophageal tissue revealed strong positive selection for clones carrying mutations in 14 cancer-related genes, with tens to hundreds of mutant clones per square centimeter. In middle-aged and older individuals, a large portion of the esophageal epithelium was occupied by clones harboring cancer-associated mutations, particularly in NOTCH1 (12%–80%) and TP53 (2%–37%). Surprisingly, NOTCH1 mutations were more prevalent in normal esophageal tissue than in esophageal cancers. These findings suggest that age-related clonal expansion is widespread in normal tissue and may influence cancer risk and development (Martincorena et al., 2018).

### 5.2 Cellular senescence and cancer progression

Cellular senescence is a prominent hallmark of aging, characterized by a stable arrest of cell proliferation. In cancer, both aging and cellular senescence involve complex mechanisms that determine cell fate. Senescence plays a dual role in cancer, acting as both a tumor suppressor and promoter, particularly influencing therapeutic responses.

Recent studies have revealed that senescence exerts paradoxical effects in EC, depending on the cellular context and temporal stage—it can either suppress or promote tumor progression. On one hand, during the early stages of ESCC development, senescence acts as a barrier to malignant transformation by preventing aberrantly proliferating cells from further division. However, for tumors to progress, cancer cells must evade or disable this checkpoint. RBM4 was found helping ESCC cells overcome senescence, particularly that triggered by oncogenes (like H-RAS) or chemotherapy (e.g., doxorubicin), thus facilitating unchecked proliferation and metabolic reprogramming towards glutamine metabolism. Targeting this escape mechanism offers a novel strategy to re-sensitize tumors to therapy by exploiting their metabolic dependencies (Chen L. et al., 2023). Additionally, SOX4 is upregulated in ESCC and suppresses senescence, thereby promoting tumor progression and resistance to chemotherapy-induced senescence, again highlighting the presence of a potential senescent microenvironment in EC (Han et al., 2016). Similarly, MYC maintains the self-renewal and undifferentiated state of esophageal epithelial basal cells, suppressing cellular senescence and linking stemness to senescence resistance in esophageal tissue (Hishida et al., 2022).

**FIGURE 1:**
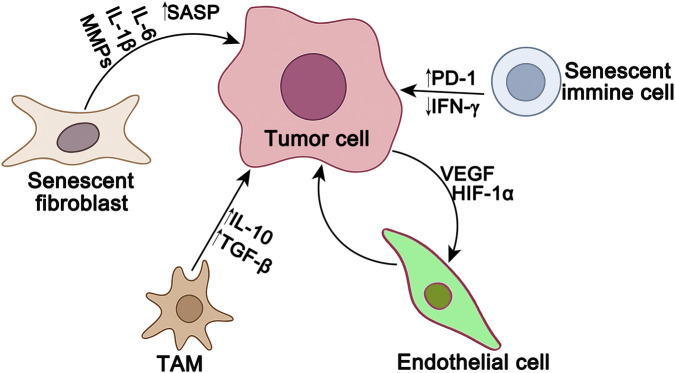
Aging-associated tumor microenvironment. Senescent fibroblasts secrete various cytokines (such as IL-6, IL-1β, and MMPs) to form a senescence-associated secretory phenotype (SASP), which promotes tumor cell growth and invasion. Senescent immune cells upregulate PD-1 and suppress the secretion of immune effector IFN-γ, leading to immune dysfunction and immune evasion. Endothelial cells, under the influence of tumor-derived VEGF and HIF-1α, promote angiogenesis to supply nutrients and oxygen to the tumor. Tumor-associated macrophages (TAMs) secrete immunosuppressive cytokines such as IL-10 and TGF-β, thereby suppressing anti-tumor immune responses.

On the other hand, in precancerous lesions and the TME, senescent cells promote tumor progression through the secretion of various senescence-associated secretory phenotype (SASP) factors (Figure 1). Although senescent cells are growth-arrested, senescent fibroblasts release a range of cytokines—such as IL-6, IL-1β, and MMPs—that profoundly reshape the microenvironment and promote tumor cell proliferation and invasion. In the hypoxic TME of ESCC, a distinct fibroblast subpopulation termed hypoxia-induced senescent cancer-associated fibroblasts (hsCAFs) has been identified; these cells secrete insulin-like growth factor 1 (IGF1) to promote tumor stemness and chemoresistance, underscoring hsCAFs as promising therapeutic targets (Ou et al., 2025). Furthermore, a recent study identified a senescence-related fibroblast gene signature—including GEM, SLC2A6, CXCL14, STX11, EFHD2, PTX3, and HCK—that predicts poor prognosis and reduced immunotherapy response in ESCC, suggesting that senescent fibroblasts play a pro-tumorigenic role and may serve as potential therapeutic targets (Zhang H. et al., 2024). Interestingly, metformin has been shown to suppress EC progression through radiation-induced cellular senescence of CAFs (Demaria et al., 2017; Sugimoto et al., 2024).

### 5.3 Age-related immune system changes

Senescent immune cells also contribute to tumor progression. The accumulation of senescent immune cells, particularly T cells and macrophages, creates an immunosuppressive environment that facilitates tumor escape and progression, especially in older patients who are highly susceptible to aging related cancer (Wu et al., 2024). These cells upregulate PD-1 expression, leading to reduced secretion of the immune effector IFN-γ, which results in immune dysfunction and escape. Meanwhile, tumor cell-derived VEGF and HIF-1α promote angiogenesis through endothelial activation, supplying oxygen and nutrients to the tumor. Tumor-associated macrophages secrete immunosuppressive cytokines such as IL-10 and TGF-β, further suppressing anti-tumor immune response (Qiu et al., 2022). Within the tumor microenvironment, factors such as glucose competition, oncogenic stress, and chronic inflammation further drive immune cell senescence, thereby promoting tumor growth and immune evasion. However, the impact of immunosenescence on the response to immune checkpoint inhibitors remains unclear, highlighting the need for age-specific immunotherapeutic strategies in esophageal cancer (Lian et al., 2020). Together, these findings suggest that senescence reshapes the tumor microenvironment to favor esophageal cancer growth, invasion, and immune evasion (Figure 1).

### 5.4 Aging and metabolic reprogramming

Cellular senescence is a state of permanent cell cycle arrest, yet these cells remain highly metabolically active. Senescence signals suppress the p53 pathway and enhance mTOR signaling, upregulating glycolytic enzymes (HK2, PKM2) and promoting the conversion of glucose to lactate, while supporting SASP secretion and sustained signaling. The imbalance between p53 and mTOR pathways also promotes GLS expression, enhancing glutamine metabolism. Senescence signals further inhibit AMPK signaling, relieving repression of lipid synthesis, and activate SREBP1 to drive FASN transcription and fatty acid synthesis. Overall, senescence drives enhanced glycolysis, glutamine metabolism, and lipid synthesis through dysregulation of energy-sensing pathways such as mTOR, AMPK, SIRT1, and p53, providing energy and biosynthetic substrates for senescent cells and maintaining inflammatory SASP secretion and metabolic homeostasis (Frasca et al., 2020; Wiley and Campisi, 2021).

### 5.5 Aging as a prognostic factor and therapeutic target in esophageal cancer

Cellular senescence is a prominent feature of the aging process and contributes to accelerated aging phenotypes observed among cancer survivors, including patients with esophageal cancer (Wang S. et al., 2024). This persistent senescence process may continue for years after diagnosis and treatment, significantly affecting long-term survival and quality of life (Dale et al., 2023). In EC, recent studies have developed prognostic models based on senescence-related gene expression, which have shown strong correlations with immune infiltration and treatment outcomes (Zheng et al., 2023).

Preclinical models, including organoids and patient-derived tumor models, offer valuable platforms to study senescence in esophageal cancer. Organoid models recapitulate the three-dimensional tissue architecture and multicellular interactions of esophageal tissues, enabling investigation of SASP-mediated effects on tumor growth, invasion, EMT, metabolic reprogramming, and therapy resistance. Patient-derived models, such as PDXs or iPSC-derived esophageal tissues, preserve individual genetic and epigenetic backgrounds, allowing exploration of patient-specific senescence dynamics and personalized therapeutic responses. Together, these models provide physiologically relevant and patient-specific systems to dissect the mechanisms of senescence-associated esophageal cancer progression and therapy resistance, although challenges remain in maintaining long-term stability and faithfully reproducing *in vivo* aging phenotypes.

The senescence marker p16 can serve as an early tumor-suppressive and senescence indicator during esophageal epithelial progression, but its epigenetic inactivation (e.g., promoter methylation) or therapy-induced re-expression is also associated with tumorigenesis (Domen et al., 2022). SA-β-Gal remains a classical functional marker of senescence (pH 6.0 enzymatic staining) and is widely applied in ESCC and tumor-associated fibroblasts to confirm chemotherapy-, hypoxia-, or radiotherapy-induced senescence and to investigate how the SASP remodels the tumor microenvironment (Amor et al., 2024; Ou et al., 2025). Loss of p16 function is one of the early molecular events in epithelial transformation, whereas its re-expression in post-treatment samples often indicates therapy-induced senescence (TIS). Chemotherapy and radiotherapy can induce senescence in both cancer and stromal cells (as indicated by increased SA-β-Gal activity and p16 expression), leading to transient growth arrest of tumor cells. However, the long-term persistence of senescent cells and their SASP secretion may promote residual tumor growth, recurrence, or therapy resistance. Therefore, TIS detection (via p16 and SA-β-Gal) represents a valuable approach for prognostic assessment and the development of senotherapeutics (senescent cell–targeting therapies) (Zhang D. et al., 2024; Yang et al., 2025). Overall, p16 and SA-β-Gal are central functional markers in esophageal cancer senescence research, yet their biological implications are complex. Integrating these markers with SASP profiles, epigenetic signatures, and microenvironmental indicators in longitudinal clinical samples is a key direction for future studies.

**FIGURE 2:**
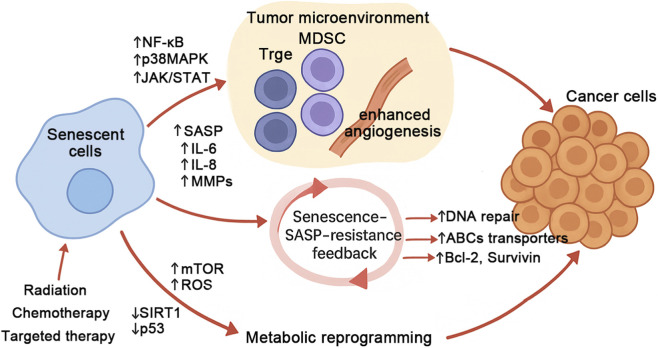
Aging-driven model of therapy resistance in cancer. Cellular senescence enhances cancer therapy resistance through the secretion of senescence-associated secretory phenotype (SASP) factors, such as IL-6, IL-8, and MMPs. These factors activate NF-κB, p38MAPK, and JAK/STAT signaling pathways, leading to remodeling of the tumor microenvironment, characterized by an increase in immunosuppressive cells (Tregs, MDSCs) and enhanced angiogenesis. SASP signaling promotes epithelial–mesenchymal transition (EMT) in cancer cells, improves DNA repair capacity, upregulates drug efflux transporters (ABC family), and activates anti-apoptotic pathways (Bcl-2, Survivin). Senescence drives metabolic reprogramming and maintains inflammatory SASP secretion and metabolic stability by dysregulating energy-sensing pathways such as mTOR, p53, and SIRT1. Radiotherapy, chemotherapy, and targeted therapy can further induce cellular senescence and SASP production, establishing a senescence–SASP–resistance feedback loop that reinforces therapy resistance and tumor progression.

Senescence is typically a response to stress, such as oncogene activation, and is marked by irreversible proliferation arrest (Gorgoulis et al., 2019). However, despite their stable growth arrest, senescent cells display metabolic reprogramming that closely resembles cancer metabolism (Kim et al., 2024). This metabolic overlap between senescent and cancer cells presents a promising therapeutic avenue, targeting these altered pathways could offer novel strategies for esophageal cancer treatment (Zhu et al., 2014a; Yamauchi and Takahashi, 2024). Cellular senescence enhances cancer therapy resistance through the secretion of SASP factors, such as IL-6, IL-8, and MMPs. These factors activate NF-κB, p38MAPK, and JAK/STAT signaling pathways, leading to remodeling of the tumor microenvironment, characterized by an increase in immunosuppressive cells (Tregs, MDSCs) and enhanced angiogenesis (Gao et al., 2025). SASP signaling promotes EMT in cancer cells, improves DNA repair capacity, upregulates drug efflux transporters (ABC family), and activates anti-apoptotic pathways (Bcl-2, Survivin). Senescence drives metabolic reprogramming and maintains inflammatory SASP secretion and metabolic stability by dysregulating energy-sensing pathways such as mTOR, p53, and SIRT1 (Frasca et al., 2020; Wiley and Campisi, 2021). Radiotherapy, chemotherapy, and targeted therapy can further induce cellular senescence and SASP production, establishing a senescence–SASP–resistance feedback loop that reinforces therapy resistance and tumor progression (Figure 2). Immune senescence has emerged as a major obstacle to effective cancer immunotherapy (Liu et al., 2024). Senescent T cells show reduced responsiveness to PD-1 blockade, impairing effector-mediated tumor clearance (Maggiorani et al., 2024). Meanwhile, senescent or therapy-induced senescent cells can transcriptionally and post-translationally upregulate PD-L1 through increased glycosylation, further suppressing antitumor immunity (Hwang et al., 2025). SASP release transiently activates immune responses but chronic exposure leads to immunosuppression, T-cell exhaustion, and the recruitment of MDSCs and Tregs, which collectively promote tumor progression (Gao et al., 2025). Immune senescence is also characterized by mitochondrial dysfunction and reduced glycolytic capacity, limiting effector and memory T-cell functions under tumor metabolic stress. Moreover, SASP–CAF metabolic crosstalk provides additional nutrients and support to cancer cells, indirectly reducing immunotherapy efficacy (Liu et al., 2024). In summary, immune senescence suppresses T-cell activity, expands immunosuppressive cell populations, and upregulates inhibitory ligands, persistently reshaping the tumor immune microenvironment and diminishing responses to immune checkpoint inhibitors. Potential intervention strategies include restoring T-cell metabolism and function, clearing or inhibiting senescent cells, targeting SASP components, and combining immunotherapy with metabolic or senotherapeutic approaches.

Currently, clinical trials targeting senescence are actively progressing, particularly among elderly or frail populations. The combination of Dasatinib and Quercetin (D + Q) is one of the first senolytic regimens tested in humans. Preclinical studies in mice have shown that D + Q effectively clears senescent cells, improves physiological function, and extends lifespan. However, human trials still face major challenges such as endpoint definition, dosage optimization, safety evaluation, and the standardization of senescence biomarkers. In oncology, early studies suggest that senotherapy may improve treatment tolerance, reduce relapse risk, and enhance long-term health outcomes, though current evidence remains preliminary and requires further validation in clinical settings (Wissler-Gerdes et al., 2021; Balducci et al., 2024; Farr et al., 2024).

## 6 Conclusion and future perspectives

Esophageal carcinoma remains a significant burden, especially in older adults. The convergence of aging-related changes with genetic instability and metabolic dysregulation defines a complex and heterogeneous tumor ecosystem. Our review outlines several aging-associated factors that influence EC progression, including clonal hematopoiesis, senescent fibroblasts, metabolic competition, and immune evasion.

Future research should prioritize developing therapies targeting senescent cells and their secretome (SASP), leverage single-cell and spatial technologies to dissect TME heterogeneity in aged patients and embrace AI-assisted computational modeling to identify vulnerabilities linked to aging. Through these efforts, the vision of the incorporation of aging biomarkers in clinical and translational EC studies of each patient for early diagnosis, can move closer to reality.

Aging is an underappreciated axis in EC pathogenesis. Deeper understanding of this dimension will support more effective and individualized interventions for older patients.
